# The Role of Health Disparities and Socioeconomic Status in Emergent Gastrointestinal Procedures

**DOI:** 10.1089/heq.2020.0141

**Published:** 2021-04-30

**Authors:** Eve May, Kristin O. Brown, Edward Gracely, Gisele Podkameni, Linda Franklin, Harpreet Pall

**Affiliations:** ^1^ Department of Gastroenterology, Hepatology, and Nutrition, Children's National Hospital, Washington, District of Columbia, USA.; ^2^ Section of Gastroenterology, Hepatology, and Nutrition, St. Christopher's Hospital for Children, Philadelphia, Pennsylvania, USA.; ^3^ Department of Pediatrics, Drexel University College of Medicine, Philadelphia, Pennsylvania, USA.; ^4^ Family, Community, and Preventive Medicine, Drexel University College of Medicine, Philadelphia, Pennsylvania, USA.; ^5^ Department of Pediatrics, Hackensack Meridian School of Medicine, Nutley, New Jersey, USA.; ^6^ Department of Pediatrics, K. Hovnanian Children's Hospital at Jersey Shore University Medical Center, Neptune, New Jersey, USA.

**Keywords:** pediatric gastroenterology, pediatric endoscopy, health disparities

## Abstract

**Objectives:** There is limited data describing the role of health disparity factors and socioeconomic status (SES) on emergent versus nonemergent gastrointestinal (GI) procedures within pediatrics. We aimed to characterize risk factors and determine the role of SES on emergent versus nonemergent GI care. We hypothesized that patients with lower SES incur higher risk of having emergent procedures performed.

**Methods:** Retrospective chart review was performed between 2012 and 2016, with 2556 patient records reviewed. Demographic data and SES categories were determined. The majority of emergent procedures were performed on an inpatient basis. Health disparity factors analyzed included age, gender, insurance type, race, language, and SES using census tracts. Logistic regression analyses and paired t-tests were utilized.

**Results:** Two hundred eighty-six (11.2%) patients had emergent GI procedures performed. Logistic regression (odds ratio [OR], confidence interval (95% CI)] showed patients from 6–11 to 12–17 years of age were less likely to seek emergent care than the youngest group [0.47, 0.33–0.66 and 0.61, 0.45–0.84]. Patients with Medicaid insurance [1.68, 1.27–2.26], African American or “other” race [2.07, 1.48–2.90 and 2.43, 1.77–3.36, respectively], as well as “other” language [2.1, 1.14–3.99] more often sought emergent care. Using geocoded data, we found that as SES increases by 1, emergent risk for procedures decreased by 2.9% (OR 0.97, *p*=0.045).

**Conclusions:** Children with lower SES, at extremes of age (<5, >18 years), non-English or Spanish speaking and with Medicaid insurance are at higher risk of undergoing emergent GI procedures. This study gives us an opportunity to plan targeted interventions to improve access and quality of care.

## Introduction

Within the field of pediatric gastroenterology, there is limited data comparing annual costs of care for gastrointestinal (GI) conditions across inpatient and outpatient settings. It has been reported that increased use of primary care decreases hospitalization costs and improved quality of health care delivery has been associated with decreased cost of inpatient procedures.^1,2^ Inequities within access to care, health service utilization, and outcomes exist and have been studied in a multitude of settings and patient populations. Within the adult GI population, Park et al. noted that non-Caucasian, higher poverty-level and underinsured patients were more likely to use inpatient care for services and treatment of inflammatory bowel disease as compared with counterparts with similar disease processes.^3^

The literature regarding how health disparities affect the utilization of pediatric gastroenterology resources and procedures is limited. A recent study from our institution identified various factors associated with higher use of emergent GI procedures, including age <5 and >18 years, African American, or non-Caucasian race, male gender, Medicaid insurance, and Spanish-speaking language.^4^ While this study identified previously undescribed associations between health disparities and emergent GI procedures, there are many unanswered questions regarding how the role of socioeconomic status (SES), specifically poverty, may drive the utilization of these unplanned procedures. With the current study, we hope to expand upon this knowledge base by stratifying SES within our population by way of geocoding.

Geocoding or geospatial mapping enables us to not only identify trends at the population level, but also identify high-risk patient populations. Within the GI literature, geocoding has primarily focused on colorectal cancer screening within the adult population.^5,6^ To date, there are no reports utilizing geospatial mapping to study social health disparities within the realm of pediatric gastroenterology. We intended to use geocoded health data to identify high-risk and vulnerable populations within our community. We also intended to further identify various factors and social determinants of health that may drive the utilization of unplanned GI procedures.

We hypothesized that patients with lower SES will have a higher risk of having GI procedures performed on an urgent or emergent basis as compared with those with higher SES. This study intends to identify and characterize health disparities to reduce barriers to health in our vulnerable and underserved pediatric population.

## Methods

This study was approved by the Institutional Review Board at Drexel University College of Medicine. The study was conducted at St. Christopher's Hospital for Children, a tertiary care academic pediatric medical center in north Philadelphia, Pennsylvania. St. Christopher's Hospital serves a diverse patient population with a significant proportion of patients from low socioeconomic backgrounds. Through this study, we examined the impact of health disparity factors on urgent vs. nonurgent GI procedures as well as the role that deprivation indices or SES has upon likelihood of urgent GI procedure utilization.

Urgent procedures were defined as any inpatient GI procedure performed in an urgent manner through nonelective inpatient care. We did not distinguish between emergent procedures as needing to be performed within 2 h of presentation as compared with urgent procedures, which are to be performed within 24 h of presentation.^7^ We did not assess time between presentation to care with timing of procedure for our patients to distinguish urgent versus emergent procedures so these two terms will be used interchangeably throughout this study. For example, patients admitted for upper endoscopy for bleeding control were classified as urgent or emergent. The decision to undergo a procedure was determined at the hospital at the time of admission. All classified as urgent or emergent procedures occurred in the inpatient setting. Nonemergent procedures were defined as a GI procedure performed in elective manner through outpatient scheduling. Patients seen in the clinic but electively scheduled for an inpatient procedure were classified as undergoing a nonemergent procedure.

Our patients were between the ages of 0 and 21 years and underwent various emergent and nonemergent GI procedures at our institution between January 2012 and December 2016. Data were collected using the hospital's billing database and individual chart review. Procedures were based on physician coding (CPT code) and ICD-9 and ICD-10 codes were recorded for the patients included in this study. We excluded patients who were seen for procedures deemed nonemergent, such as pH impedance probe, percutaneous endoscopic gastrostomy placement, and manometry studies, as these studies are largely performed for nonemergent reasons, even if performed in the inpatient setting. Endoscopic retrograde cholangiopancreatography were excluded from this study as they were not performed at our pediatric center. We excluded duplicate encounters for patients with more than one emergent procedure, in which case only the first procedure was included in our data set. Patients over 21 years of age were excluded.

Health disparity factors collected from patient records included age, gender, insurance type, race, and language. Age and insurance were evaluated at the time of patient procedure. Age was classified into four distinct groups: 0 to 5, 6 to 11, 12 to 17, and 18 to 21 years to represent early childhood, middle childhood, adolescence, and young adulthood, respectively. Gender was classified as either male or female. Type of insurance was classified as Medicaid or commercial insurance. Only one patient was uninsured and excluded from analysis. Race was self-reported and classified into four groups: African American, Hispanic, Caucasian, and other. Finally, primary language spoken by parent was identified as Spanish English, or other. In our medical record system, Hispanic was defined as both race and ethnicity although policy typically defines Hispanic as an ethnicity rather than a racial group.

In addition to collecting patients' health disparity factors, we recorded the patient's zip code on the day of service (DOS), which was utilized to determine scores for SES as linked to the individual's census tract. The DOS is a vital component to this study since SES can change over time if the economic profile of a census tract has changed. Deprivation scores, a focus of deprivation measures used to understand health inequalities, were determined based on SES utilizing principal factor analysis of 16 measures from the U.S. Census, including housing, stability (living in same hours 5 years prior), education (high school vs. college), employment (unemployed, labor force participation), occupation, and income/wealth. The deprivation score for SES then creates a weighted score for neighborhood deprivation for each census tract.

### Data analysis

From a statistical standpoint, our goal was to identify health disparities between the two groups of patients undergoing emergent versus nonemergent procedures. Statistics were performed using the Statistical Package for Social Sciences (SPSS™) to determine which health disparity variables were independent predictors of having an emergent procedure, using a logistic regression analysis. Preliminary univariate logistic regressions were run followed by a multiple logistic regression holding age, primary payer, and race constant. In the logistic regression model, all variables were nominal and all variables were either dichotomous (notably gender and insurance type) or were converted to dummy coded variables. For the latter, the reference groups were age 0–5 years (for age), Commercial (for insurance type), and Caucasian (for race/ethnicity). All reference groups had enough subjects for stability.

The various deprivation indices using SES scores for each patient were calculated by Drexel University's United Health Collaborative (UHC), a group that works to identify and improve population health and lessen health inequities. We transferred each patient's zip code and DOS to UHC through REDCap™, a secure web application, enabling us to protect patient identifiers. UHC utilized geocoding techniques through 2012–2016 census data from the American Community Survey, which includes information on housing, employment, education level, and income for each census tract to calculate deprivation indices associated with zip codes matched to each patient's residence. Factor scoring was created by analysis performed by Diez Roux and the scale represents a basic SES score as one variable scale, using the variables listed above.^8^ The SES measure used is a calculated factor score that in our data ranged from −12 to 14.8, with a mean−2.1, median −2.2, and SD 5.5. Once geocoded data were given an SES score, logistic regression was run with comparison to initial health disparity variables.

## Results

We performed a retrospective chart review of the electronic medical records during our study period, where we found that of 3830 patients who had GI procedures performed, 2556 met inclusion criteria and were included in the study. Of these patients, 286 (11.2%) had an emergent GI procedure performed (Table 1). As shown in Table 1, the overall study population was most commonly between 12 and 17 years of age (50.8%), female (57.3%), insured by Medicaid (67%), Caucasian (40.6%), and English speaking (90.6%).

**Table 1.** Patient Demographics

		Total population, *n*=2556	Emergent procedure group, *n*=286	Nonemergent procedure group, *n*=2270
	Health disparity factors	*n* (% of total)	*n* (% of demographic group)	*n* (% of demographic group)
Age	0–5 years	532 (20.8)	90 (16.9)	442 (83.1)
	6–11 years	741 (28.9)	59 (7.9)	682 (92.0)
	12–17 years	1147 (50.8)	110 (9.6)	1037 (90.4)
	18–21 years	136 (6.0)	27 (19.9)	109 (80.1)
Gender	Female	1293 (57.3)	133 (10.3)	1160 (89.7)
	Male	1263 (42.7)	153 (12.1)	1110 (87.9)
Health insurance	Commercial	843 (32.9)	67 (7.9)	776 (92.1)
	Medicaid	1713 (67.0)	219 (12.8)	1494 (87.2)
Race	African American	475 (18.5)	74 (15.6)	401 (84.4)
	Hispanic	542 (21.2)	40 (7.4)	502 (92.6)
	Other	500 (19.6)	88 (17.6)	412 (82.4)
	White	1039 (40.6)	84 (8.1)	955 (91.9)
Language	English	2315 (90.6)	255 (11.0)	2060 (89.0)
	Spanish	178 (7.0)	18 (10.1)	160 (89.9)
	Other	63 (2.5)	13 (20.6)	50 (79.4)

Based on ICD 9 and ICD 10 codes, the most common reasons for procedures in the urgent and emergent groups were generalized abdominal pain, GI tract hemorrhage, and hematemesis. This compares to the most common ICD 9 and ICD 10 codes in the nonemergent groups including generalized abdominal pain and gastroesophageal reflux without esophagitis. A total of six procedure types were identified in our study population and all procedures were seen in both emergent and nonemergent groups and included rectal biopsy, liver biopsy, colonoscopy, sigmoidoscopy, upper endoscopy, and capsule endoscopy. Comparing distributions of procedures between groups, we found that the most common procedure in both groups was upper endoscopy for the emergent procedure group (90.6%) as compared with 92.3% for the nonemergent group.

Using univariate logistic regression, age (*p*<0.001), race (*p*<0.001), and insurance type (*p*<0.001) were significant predictors of emergent status, as shown in Table 2. As seen in that table, the two middle groups in the age range had odds ratios (ORs) for emergent that were much below 1 compared with the youngest children (the reference group), whereas the oldest group did not, and had, in fact, a slightly higher rate of emergent procedures (Table 1). Note that the confidence interval (95% CI) for the highest age group is nonoverlapping with the two middle groups, indicating that the OR in the oldest group is strongly significantly higher (although logistic regression does not directly test that). Patients on Medicaid had higher odds of being emergent than those with commercial insurance. Both African American and Other races were associated with higher odds of being emergent compared with Caucasians.

**Table 2.** Univariate Potential Predictors of Emergent Procedures

Health disparity variable	Odds ratio of emergent procedure	95% confidence interval	*p*
Age, years			<0.001
0–5^*^	—	—	—
6–11	0.42	0.30–0.60	<0.001
12–17	0.52	0.39–0.70	<0.001
18–21	1.22	0.76–1.99	0.42
Gender			
Male^*^	—	—	—
Female	0.83	0.65–1.06	0.143
Insurance			
Commercial^*^	—	—	—
Medicaid	1.68	1.27–2.26	<0.001
Race			<0.001
Caucasian^*^	—	—	—
African American	2.07	1.48–2.9	<0.001
Hispanic	0.93	0.63–1.37	0.709
Other	2.43	1.77–3.36	<0.001
Language			0.058
English^*^	—	—	—
Spanish	0.91	0.55–1.5	0.91
Other	2.1	1.14–3.99	0.02

^*^ Indicates reference value.

**FIG. 1.**
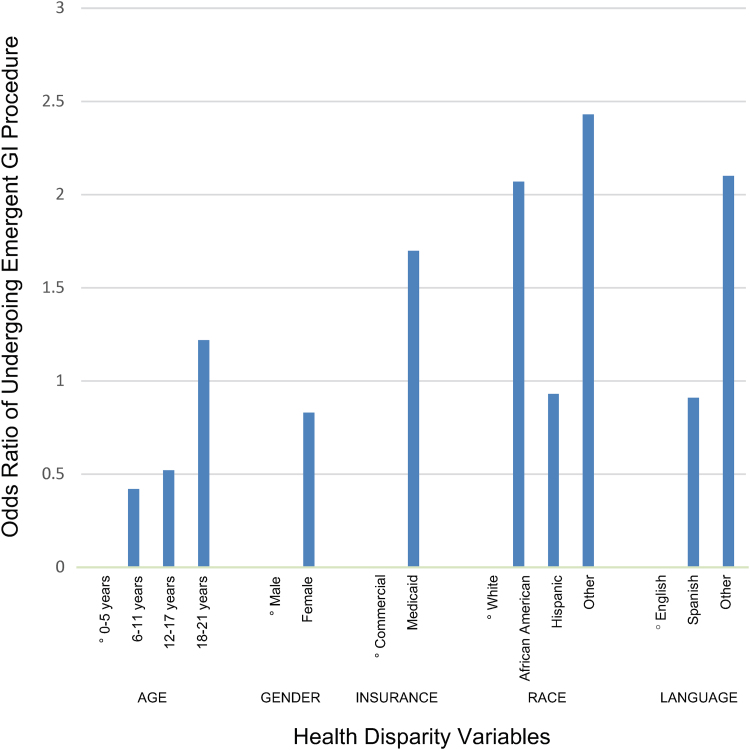
Rate of emergent gastrointestinal procedures given health disparity factors. Logistic regression identified odds of undergoing emergent gastrointestinal procedures. °Indicates health disparity factor category that served as reference group for statistical analysis. GI, gastrointestinal.

Figure 1 graphically depicts the odds of undergoing an emergent procedure for each variable outcome. The oldest and youngest ages, Medicaid insurance, African American or other race, and other language were all found to be at a higher risk of having an emergent procedure performed as compared with their counterparts (Fig. 1).

A total of 300 different zip codes with 807 unique census tracts were included in this study. Geocoded data were linked to the patient's SES and compared between both emergent and nonemergent procedure groups. Using an unpaired *t*-test, emergent patients had lower SES than nonemergent (*p*<0.001). A univariate logistic regression returned an OR of 0.97 such that every increase of 1 in SES (on this particular scale) multiplies the odds of being an emergent case by 0.97. By exponential calculations, an increase of 5 (about 1 SD or a bit less) in SES would multiply the odds of emergent procedure by 0.79.

We wanted to next assess what was driving these relationships, especially race and SES. It was decided to exclude gender (which had *p*=0.14 for association with emergent procedure and was unlikely to confound associations involving race) and language (with *p*=0.058 but with only the ambiguous “other” category significant). Logistic regression with multivariate analysis was performed using payor, race, and age group as well as SES (Table 3). Again, we found that patients with higher SES scores were less likely to have a procedure performed on an emergent basis (OR 0.97, *p*=0.045). In this analysis, we also found that patients were more likely to have emergent procedure who were African American or other race (OR 1.47, OR 1.82, respectively), and again the two middle age groups had lower odds of an emergent procedure than the youngest group (reference) and the oldest (by virtue of CI comparison).

**Table 3.** Multivariate Analysis of Potential predictors of Emergent Procedures, including Socioeconomic Status

Health disparity variable	Odds ratio of emergent procedure	95% confidence interval	*p*
SES	0.97	0.94–1.00	0.045
Age, years			<0.001
0–5^*^	—	—	—
6–11	0.46	0.33–0.66	<0.001
12–17	0.61	0.45–0.84	0.002
18–21	1.31	0.80–2.13	0.278
Insurance			
Commercial^*^	—	—	—
Medicaid	1.33	0.96–1.85	0.086
Race			<0.001
Caucasian^*^	—	—	—
African American	1.47	1.02–2.13	0.041
Hispanic	0.65	0.42–1.01	0.055
Other	1.82	1.28–2.59	<0.001

^*^ Indicates reference value; SES, socioeconomic status.

## Discussion

Our study found that children with lower SES, at extremes of age (<5, >18 years), Spanish or non-English, and with Medicaid insurance, are at higher risk of undergoing emergent GI procedures. First, we noted that most GI procedures were performed in the outpatient setting on a nonemergent basis (89%). This is consistent with previous reports from the Pediatric Endoscopy Database System-Clinical Outcomes Research Initiative (PEDSCORI), which showed 85% of GI procedures were delivered in the outpatient setting.^9^ The most common procedures performed in both settings was upper endoscopy. Based on ICD 9 and ICD 10 codes, abdominal pain was the most likely indication for endoscopy in both the emergent and nonemergent procedure groups. This is consistent with many reports noting that chronic abdominal pain is the most common indication for upper endoscopy in the pediatric population.^10,11^ In contrast, endoscopy for GI hemorrhage was limited to only the emergent procedure group which is understandable given the possibility of clinical deterioration should this not be addressed in an acute setting.

With regards to health disparity variables, our study found different utilization patterns for GI procedures with regards to age, language spoken, insurance type and race. Patients most likely to undergo emergent GI procedures include patients in the older and younger age ranges for pediatric cases, those of African American race, having Medicaid insurance, and speaking neither English nor Spanish. These findings were largely consistent with a previous study performed at our institution, where patients >18 years old, African American race and Medicaid insurance were at higher risk to have a procedure in the emergent setting. Further research is needed to understand why these age groups have higher rates of emergent procedures. It may be that there is parental anxiety associated with younger children and a number of comorbid conditions, including developmental delay, in older children. Furthermore, it is interesting that there is an incongruence with predominant age ranges for these two studies as we do not expect that our population changed dramatically over a short time period. It is possible that there are additional variables present that were not accounted for this in study, or possibly including SES in our statistical analysis made significant variables less significant.

We did not find a predilection for one gender to have an emergent procedure performed over another. This parallels a recent study that reported no variations between genders for rates of endoscopies in either pediatric or adult population.^9^ The youngest and oldest age groups were at higher risk of having emergent procedures (Fig. 1).

Reports have revealed racial disparities within the health care field. Our findings parallel a study that demonstrated an increased rate of admissions for GI bleeds for African American patients as compared with Caucasian patients.^12^ Similarly, patients who predominantly spoke languages other than English or Spanish were at a two-fold risk of having an emergent GI procedure performed. It has been reported that patients who required an interpreter were more likely to have an inpatient procedure as compared with English-speaking patients.^13^ Alternatively, it is possible that language barriers may affect how providers deliver care. Patients with Medicaid insurance had odds 1.68 times greater of having an emergent GI procedure performed, but only in the univariate analysis. Insurance type was not significant when SES was controlled for.

Utilizing census tracts helps us to gain valuable information about measures of economic deprivation.^14–17^ SES is a composite of many factors that make up a patient's health and wellness and encompasses far more than the individual health disparity variables captured in this study. Our study found that higher SES was inversely correlated with the risk of having an emergent procedure. This suggests that geospatial mapping can be utilized as a screening tool to evaluate health care disparities. Once identified, more specific health factors such as obesity, level of education, and mental health, for example, could be evaluated and correlated with geocoded data.

The strength in this study lies in its capacity to identify both individual and communities at risk of emergent GI procedures as compared with those performed in a more controlled setting. This study has pioneered the use of geocoding to stratify the pediatric gastroenterology population to identify these high-risk groups.

This study is limited in that it was performed at a single, urban institution, thereby reducing generalizability. Second, our distribution of patients was not evenly split between groups, with many procedures being performed in the nonemergent groups. Despite serving an underserved population in North Philadelphia, we had a surprising lack of diversity within our patient population as the majority of patients were English speaking with Medicaid insurance. Furthermore, this study is observational in nature, implying only correlation rather than causation for all variables evaluated.

This study could be expanded upon by looking at additional meaningful end-points contributing to lower SES such as obesity or mental health for example, which could then be targeted for interventions to improve access to care in our urban population. Further qualifying characteristics associated with drivers of unplanned GI procedures through an interview process for hospitalized patients could add significant depth to this project and could be a valuable future application of the current study.

## Conclusion

In summary, our study found that children with lower SES, at extremes of age (<5, >18 years), Spanish or non-English and with Medicaid insurance are at higher risk of undergoing emergent GI procedures as compared with their counterparts. While this study adds to the body of literature, which has found the importance of social determinants of health, additional work needs to be done to identify and address the underlying causes of poverty and help to improve access to outpatient care in our disadvantaged and at-risk groups.

## Abbreviations Used

DOS: day of service
EMR: electronic medical records
GI: gastrointestinal
PEDSCORI: Pediatric Endoscopy Database System-Clinical Outcomes Research Initiative
SES: socioeconomic status
SPSS: Statistical Package for Social Sciences
UHC: United Health Collaborative
